# Telodendrimer-Based Macromolecular Drug Design using 1,3-Dipolar Cycloaddition for Applications in Biology

**DOI:** 10.3390/molecules25040857

**Published:** 2020-02-15

**Authors:** Hossein Yazdani, Esha Kaul, Ayoob Bazgir, Dusica Maysinger, Ashok Kakkar

**Affiliations:** 1 Department of Chemistry, McGill University, 801 Sherbrooke St. West, Montréal, QC H3A 0B8, Canada; hyazdani21@gmail.com; 2 Department of Chemistry, Shahid Beheshti University G.C., Tehran 1983963113, Iran; a_bazgir@sbu.ac.ir; 3 Department of Pharmacology and Therapeutics, McGill University, 3655 Promenade Sir William Osler, Montréal, QC H3G 1Y6, Canada; esha.kaul@mail.mcgill.ca

**Keywords:** telodendrimer, macromolecular drug, 1,3-dipolar cycloaddition, glioblastoma

## Abstract

An architectural polymer containing hydrophobic isoxazole-based dendron and hydrophilic polyethylene glycol linear tail is prepared by a combination of the robust ZnCl_2_ catalyzed alkyne-nitrile oxide 1,3-dipolar cycloaddition and esterification chemistry. This water soluble amphiphilic telodendrimer acts as a macromolecular biologically active agent and shows concentration dependent reduction of glioblastoma (U251) cell survival.

## 1. Introduction

**Figure 1:**
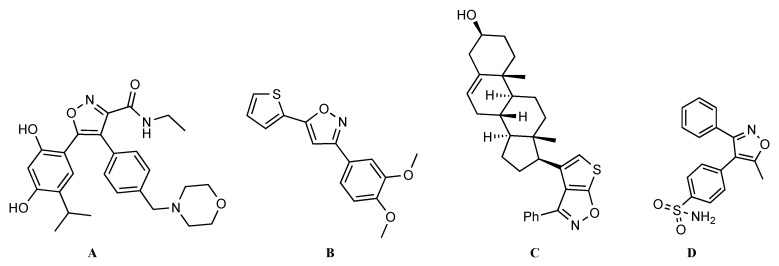
Selected examples of bioactive isoxazoles: **A**, **B** and **C**, 3,4,5-isoxazole and fused isoxazole derivatives with anticancer; and **D**, 3,4,5-isoxazole derivative with anti-inflammatory activity.

The isoxazole moiety, a five-membered heterocycle containing oxygen and nitrogen at adjacent positions, has shown interesting biological properties including anticancer [1], antimicrobial [1], antibacterial [2], anti-inflammatory [3], antiviral [4], antithrombic [5], antiapoptotic [6] and antiparasitic activity [7]. For example, NVP-AUY922 (Figure 1A) is an anticancer agent [1] and thiophen-2-yl isoxazole (Figure 1B) significantly inhibits proliferation of HeLa cells. Isoxazole with a steroid moiety (Figure 1C) has displayed considerable cytotoxicity towards tumor cell lines and sulfisoxazole, flucloxacillin, dicloxacillin, oxacillin, sulfamethoxazole and cloxacillin are some examples of marketed antimicrobial drugs bearing an isoxazole ring [8]. Valdecoxib (Figure 1D) is a nonsteroidal anti-inflammatory drug (NSAID) that was targeted towards cyclooxygenase enzymes to hinder prostaglandin biosynthesis and to reduce pain [8]. About thirty patents covering different isoxazole-based structures have been reported showing a wide array of chemical modifications and applications of such compounds [8,9,10,11,12,13,14,15,16,17,18]. Macromolecular therapeutics are increasingly proposed as nanomedicines [19] and new methodologies to prepare highly potent isoxazoles bearing nanocarriers and nanotherapeutics will likely expand the scope of their applications.

A tremendous research effort has been devoted to developing nanocarriers that could deliver active pharmaceutical agents to the sites affected by pathological changes to minimize wide body distribution and reduce undesirable side effects [20,21]. Amphiphilic linear block copolymers have been extensively explored in assembling core-corona type spherical micelle structures for drug delivery [22]. It is now well known that the overall macromolecular composition and structure play a vital role in modulating important parameters including plasma residence time and drug loading/controlled release behavior in these formulations [23]. This has led to expanding the macromolecular space from linear-to-branched-to-hyperbranched architectures [24]. Telodendrimers that are hybrids of hyperbranched dendrimers and linear polymers have offered an advantageous platform in combining individual properties of these components [25,26,27,28,29]. 

Considering the biological potency of isoxazoles, we were interested in developing synthetic methodologies to water-soluble macromolecular drugs using amphiphilic architectural polymers, in which the hydrophobic dendron segment will contain isoxazole heterocyclic rings and a linear poly(ethylene glycol) methyl ether (mPEG) tail will impart hydrophilic character. 1,3-Dipolar cycloaddition is among some of the most important reactions in organic and medicinal chemistry [30] and it has been used for the isoxazoles scaffold synthesis by the cycloaddition of nitrile oxides and alkynes [31]. To the best of our knowledge, employing 1,3-dipolar cycloaddition reaction based on nitrile oxides in synthesizing dendrons/dendrimers has not been reported. We report herein such a synthetic approach to construct an isoxazole-mPEG based architecture polymer, its behavior in an aqueous medium and an assessment of its ability to act as a macromolecular agent that can reduce or even abolish viability of glioblastoma cells.

## 2. **Results and Discussion**

### 2.1. Synthesis

**Scheme 1:**
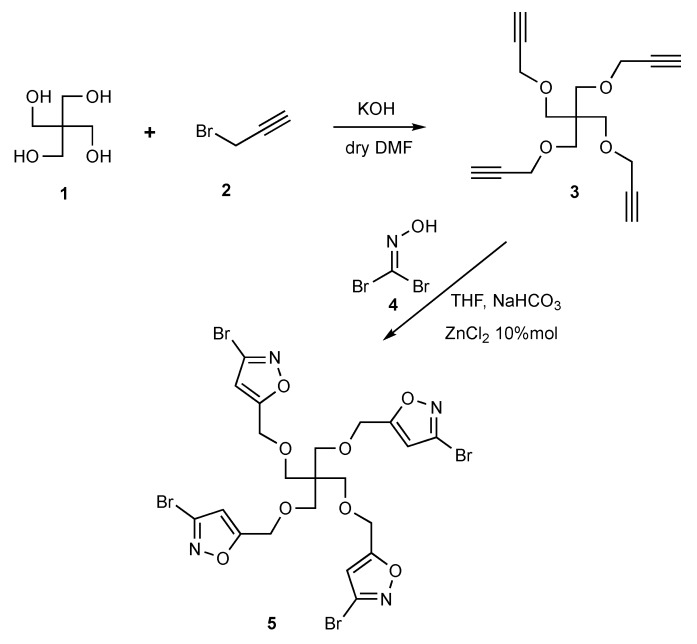
Synthesis of 5,5′-(((2,2-bis(((3-bromoisoxazol-5-yl)methoxy)methyl)propane-1,3-diyl)bis(oxy))bis(methylene))bis(3-bromoisoxazole).

We first explored the potential of 1,3-dipolar cycloaddition strategy in the synthesis of a symmetric generation 0 dendrimer. It was begun by the propargylation of pentaerythritol using propargyl bromide with potassium hydroxide in dry DMF (Scheme 1) [32], followed by the 1,3-dipolar cycloaddition on the tetrapropargylated core (**3**) using a bromonitrile oxide precursor (**4**) in THF at 45 °C for 3 h, with NaHCO_3_ as base and ZnCl_2_ as a catalyst. It afforded 5,5′-(((2,2-bis(((3-bromoisoxazol-5-yl)methoxy)methyl)propane-1,3-diyl)bis(oxy))bis(methylene))bis(3-bromoisoxazole) (**5**) in 44% isolated yield (Scheme 1) [33].

**Scheme 2:**
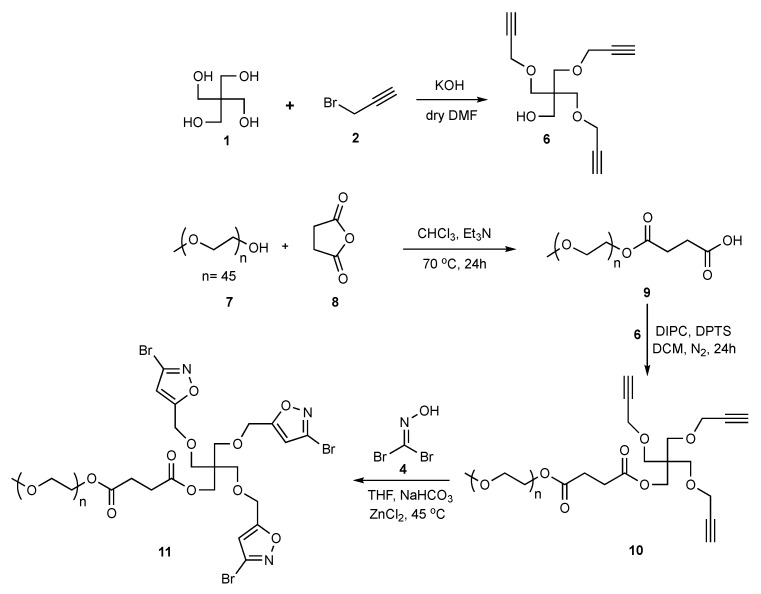
Synthesis of isoxazole-mPEG architectural polymer (**11**).

Isoxazole bearing dendrimer **5** was not soluble in common organic solvents and it prevented us from expanding the scope of dendrimer build-up or its surface functionalization to enhance its solubility and examine its biological activity. We subsequently considered designing an architectural polymer by replacing one of the isoxazole based arm with PEG. For this proposal, we first synthesized a tripropargylated pentaerythritol core by reacting pentaerythritol with 3 equivalents of propargyl bromide [28], which leaves a free OH group at the core (**6**, Scheme 2). mPEG-COOH (**9**) was synthesized by reacting mPEG_2000_ with succinic anhydride in the presence of triethylamine (Et_3_N) in CHCl_3_ at 70 °C for 24 h. The esterification reaction of mPEG-COOH (**9**) with the tripropargylated pentaerythritol core (**6**) was subsequently carried out using 1,4-dimethylpyridinium p-toluenesulfonate (DPTS) and diisopropylcarbodiimide (DIPC) in dry CH_2_Cl_2_. The desired telodendrimer with isoxazole rings was subsequently synthesized using a 1,3-dipolar cycloaddition reaction on **10**, using a bromonitrile oxide precursor (Scheme 2).

**Figure 2:**
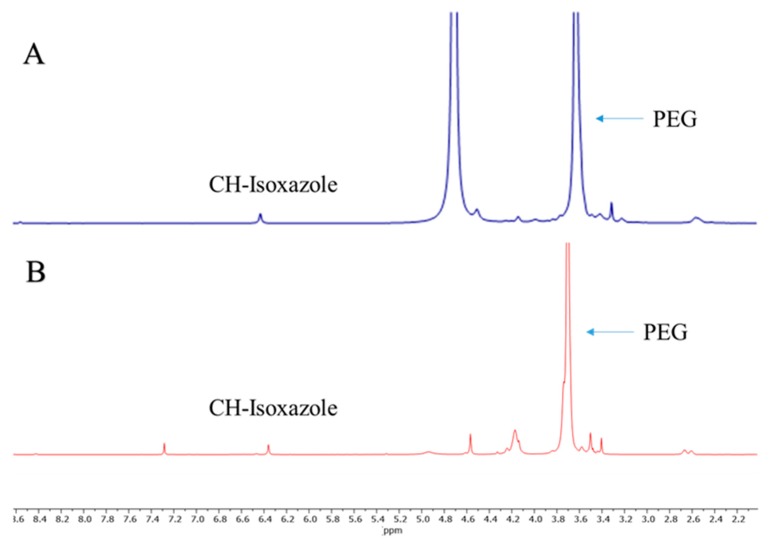
^1^H nuclear magnetic resonance (NMR) (500 MHz) spectrum of isoxazole telodendrimer in D_2_O (**A**) and CDCl_3_ (**B**).

Telodendrimer **11** was soluble in common organic solvents and it was characterized using a combination of techniques including nuclear magnetic resonance (NMR) and mass spectrometry. Its aqueous behavior was first examined using ^1^H NMR in D_2_O (Figure 2). As expected, dendron peaks become broad while those related to polyethylene glycol retained their original peak configurations. This suggests that the telodendrimer assumes a hydrophobic core/hydrophilic corona type structure in D_2_O.

**Figure 3:**
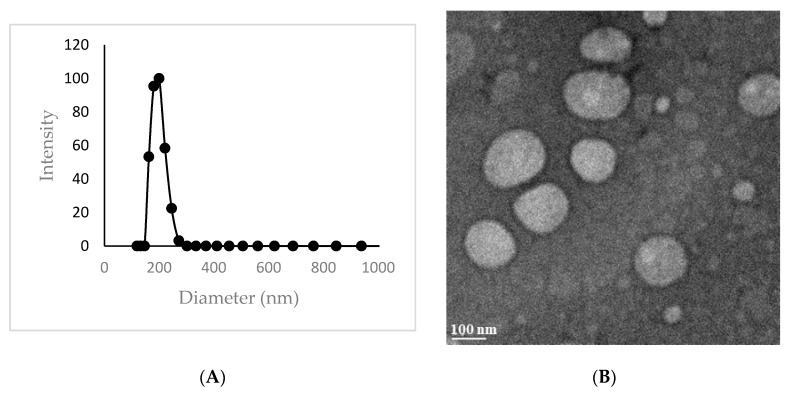
(**A**) Dynamic light scattering (DLS) size distribution and (**B**) transmission electron microscopy (TEM) image for the aqueous assemblies of isoxazole telodenderimer. The concentration of the polymeric micelle solution was 2 mg/mL for TEM study.

We examined similar self-assembly of the telodendrimer using an acetone/water co-solvent evaporation method [34,35,36]. The resulting assemblies were characterized using dynamic light scattering (DLS) and transmission electron microscopy (TEM) (Figure 3). These suggested that spherical structures were formed with a size distribution of 200 and ~60 nm, as measured by DLS and TEM respectively. As is common with solution based DLS measurements, hydrodynamic sizes of these assemblies were found to be higher as compared to TEM [37]. We were unable to obtain a critical micelle concentration of the assembled structures using pyrene encapsulation. This may suggest that pyrene does not get incorporated into the hydrophobic core due to inability of the self-assembled structures from these telodendrimers to solubilize pyrene [38]. However, using the DLS intensity method, an inflection point was measured to be at 0.35 mg/mL. This value is high and suggests that at dilutions encountered during circulation, the particles observed through DLS and TEM will be disassembled.

### 2.2. Isoxazole Telodendrimer Reduces Glioblastoma Cell Viability

**Figure 4:**
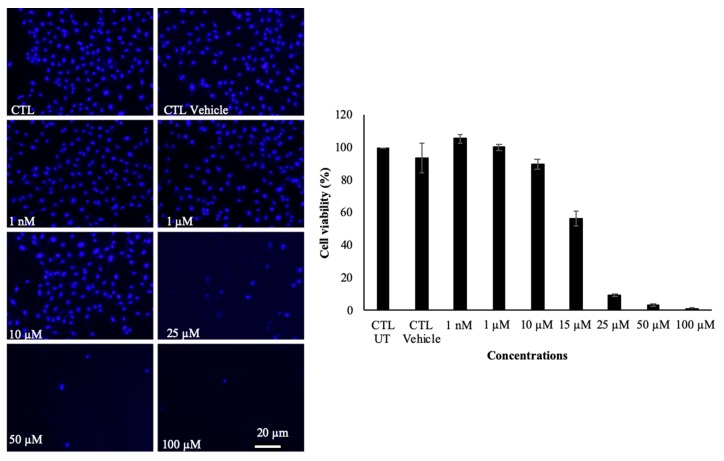
Glioblastoma cell viability studies: U251N cells were treated with aqueous formulations of the telodendrimer (1 nM, 1 µM, 10 µM, 25 µM, 50 µM and 100 µM) for up to 72 h. Cells were fixed and stained with Hoechst 33342. Vehicle indicates 15% milli-q water. More than 130 images were analyzed for each condition. Number of cells were quantified by imaging them using Leica Fluorescent Microscope.

We explored the concentration-dependent cytotoxicity of the telodendrimer using cell viability assay. Brain tumor cells (U251N) were treated with increasing concentrations of the telodendrimer, ranging from 1 nM to 100 µM and cell nuclei were counted after 24 h and 72 h. As an additional control, cells were treated with 15% milli-q (MQ) water, since the telodendrimer was diluted in milli-q water to make stock concentrations. The presence of MQ water did not affect cell viability. None of the concentrations below 10 µM caused significant cell death after 24 h and 72 h (Figure 4). However, 100 µM concentration of the telodendrimer significantly reduced the number of viable cells to 9.89% and 1.35% after 24 h and 72 h, respectively (Figure 4). Results from this study demonstrate that isoxazole-based telodendrimer acts as a macromolecular drug and exhibits good cytotoxicity towards U251N cells. Isoxazole based compounds, such as NVP-AUY922 [39], as well as Leflunomide [40] exhibit anti-tumor activity. Certain 3,5-diaryl isoxazole linked 2,3-dihydroquinazolinones are cytotoxic towards U251N cells. These compounds kill cells by disrupting microtubules and fragmenting cell nuclei [41]. Morphological abnormalities seen in U251 glioblastoma cells exposed to the telodendrimer included pyknotic nuclei, suggesting a small contribution of apoptotic cell death. Analysis of mitochondrial functions in these cells (using tetrazolium salt, an indicator of mitochondrial metabolic activity) showed reduced function of these organelles upon telodendrimer treatment. Mitochondrial functional impairment was also concentration dependent; it was most profound in > 10 μM telodendrimer concentrations and it correlated with the decline in glioblastoma cell number. We are currently investigating several modes of cell death caused by isoxazole-based telodendrimers including ferroptosis [42,43,44,45,46,47,48,49,50]. Our further aim in this direction is to explore cell death mechanism, to identify the implicated signal transduction pathways.

## 3. Materials and Methods

### 3.1. General

The NMR spectra were recorded on AV 400 and 500 MHz (Bruker, Beerlika, MA, USA) spectrometers both equipped with BBFO+ smart probes. Mass spectra analyses (HRMS, ESI) were performed on an Exactive Plus Orbitrap-API (Thermo Scientific, Waltham, MA, USA) high resolution mass spectrometer. Chemicals were purchased from Sigma Aldrich (St. Louis, MO, USA), ChemImpex International (Wood Dale, IL, USA), Alfa Aesar (Ward Hill, MA, USA), Fisher scientific (Hampton, NH, USA) and ACP Chemicals (Saint-Leonard, QC, Canada), and were used without further purification. Dry solvents were obtained from a solvent purification system. TEM samples for imaging were prepared by placing a 10 µL drop of the polymeric micelle solution (2 mg/mL) onto a carbon-coated copper grid. Excess solution was removed after 2 min using a Whatman filter paper. 10 µL of 2% uranyl acetate was then dropped onto the grid, left for 2 min and excess removed by a Whatman filter paper as before. The grid was then left to dry for an additional 20 min before imaging.

### 3.2. Synthesis

3-(3-(prop-2-yn-1-yloxy)-2,2-bis((prop-2-yn-1-yloxy)methyl)propoxy)prop-1-yne

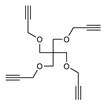



A mixture of pentaerythritol (1.00 g, 7.35 mmol) and potassium hydroxide (6.25 g, 0.112 mol) in dry DMF (25 mL) was stirred at 0 °C under nitrogen. After 5 min a solution of propargyl bromide (7.8 mL, 0.084 mol) in toluene (80%) was added drop wise and the reaction mixture was left to stir for 24 h. After completion of the reaction, water was added, and the compound was extracted with diethyl ether (3 × 5 mL) and washed with brine. The organic layer was isolated and dried over anhydrous Na_2_SO_4_. The solvent was removed under vacuum and the desired product was purified by column chromatography (hexane/EtOAc = 8.5:1.5) (Yield: 1.2 g, 57%). ^1^H NMR (400 MHz, CDCl_3_): δ_H_ (ppm) 2.41 (4H, t, ^4^*J*_HH_ = 2.4 Hz, C≡CH), 3.52 (8H, s, C-CH_2_-O), 4.12 (8H, d, ^4^*J*_HH_ = 2.4 Hz, O-CH_2_-C≡C). ^13^C{^1^H} NMR (100 MHz, CDCl_3_): δ_C_ (ppm) 44.8, 58.7, 69.0, 74.0, 80.0. 

5,5′-(((2,2-bis(((3-bromoisoxazol-5-yl)methoxy)methyl)propane-1,3-diyl)bis(oxy))bis(methylene))bis(3-bromoisoxazole).

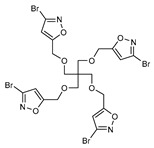



A mixture of tetrakis(2-propynyloxymethyl) methane (0.288 g, 0.001 mol), dibromoformaldoxime (1.010 g, 0.005 mol), ZnCl_2_ (40 mol%) and NaHCO_3_ (0.672 g, 0.008 mol) in THF (5 mL) was stirred at 45 °C for 3 h. The solvent was then removed under vacuum and the crude mixture was washed with H_2_O (10 mL), diethyl ether (10 mL) and then dried to afford the desired pure product (Yield: 0.340 g, 44%). ^1^H NMR (400 MHz, DMSO-*d_6_*): δ_H_ (ppm) 3.43 (8H, s, C-CH_2_-O), 4.60 (8H, s, O-CH_2_-isoxazole ring), 6.76 (4H, s, HC=C).

3-(prop-2-yn-1-yloxy)-2,2-bis((prop-2-yn-1-yloxy)methyl)propan-1-ol

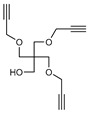



A mixture of pentaerythritol (1.00 g, 7.35 mmol) and potassium hydroxide (2.8 g, 0.05 mol) in dry dimethylformamide (DMF, 20 mL) was stirred at 0 °C under nitrogen. After 5 min a solution of propargyl bromide (4.76 g, 5.95 mL, 0.040 mol) in toluene (80%) was added drop wise to the reaction mixture. After warming to room temperature slowly, the reaction mixture was left to stir for 24 h at 40 °C under nitrogen. After completion of the reaction and cooling to room temperature, water was added to the mixture and the compound extracted with diethyl ether (3 × 5 mL) and washed with brine. The organic layers were isolated and dried over anhydrous Na_2_SO_4_. The solvent was removed under vacuum. Purification of the crude material was done by column chromatography (hexane/EtOAc = 7.5:2.5) to afford the desired product (Yield: 1.1 g, 60%). ^1^H NMR (500 MHz, CDCl_3_): δ_H_ (ppm) 2.43 (3H, t, ^4^*J*_HH_ = 2.5 Hz, C≡CH), 3.56 (6H, s, C-CH_2_-O), 3.68 (2H, s, CH_2_-OH), 4.12 (6H, d,^4^*J*_HH_ = 2.5 Hz, O-CH_2_-C≡C). ^13^C{^1^H} NMR (75 MHz, CDCl_3_): δ_C_ (ppm) 44.6, 58.7, 64.9, 70.1, 74.5, 79.6.

mPEG_2000_-COOH

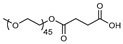



A mixture of poly(ethylene glycol) methyl ether 2000 (mPEG 2000) (3g, 1.5 mmol), succinic anhydride (0.25 g, 0.002 mol) and Et₃N (0.2 ml, 0.0015 mol) in CHCl_3_ (5 mL), was refluxed at 70 °C for 24 h. After completion of the reaction, water was added to the mixture and the compound was extracted (3 × 10 mL) with chloroform and washed with brine. The organic layer was isolated and dried over anhydrous Na_2_SO_4_. The solvent was then removed under reduced pressure and dried. The solid residue was purified further from CHCl_3_/Ether (1:5) to afford the pure product with a yield of 2.7 g, 86%. ^1^H NMR (400 MHz, CDCl_3_): δ_H_ (ppm) 2.66 (4H, m, -CH_2_-CH_2_-COOH), 3.40 (3H, s, OCH_3_), 3.11–3.85 (178 H, m, PEG), 4.28 (2H, t, ^3^*J*_HH_ = 4 Hz, -CH_2_-OCO-). ^13^C{^1^H} NMR (125 MHz, CDCl_3_): δ_C_ (ppm) 29.4, 44.9, 58.9, 63.6, 68.9, 70.5, 71.8, 172.4, 174.6.

mPEG_2000_-tripropargyl pentaerythritol

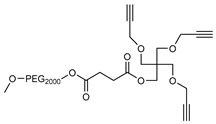



In a three-neck flask mPEG-COOH (2.1 g, 0.001 mol), tripropargyl pentaerythritol (0.25 g, 0.001 mol) and DPTS (0.279 g, 0.001 mol) were dissolved in dry CH_2_Cl_2_. A solution of diisopropylcarbodiimide (DIPC 0.176 g, 0.0014 mol) in dry CH_2_Cl_2_ was added dropwise to the flask over 1.5 h under nitrogen atmosphere. The reaction was stirred for 24 h. Water was then added and the compound extracted (2 × 10 mL) with dichloromethane. The mixture was then washed with NaHCO_3_ (5%) and extracted with CH_2_Cl_2_. After that, the organic layer was washed with brine and then dried over anhydrous Na_2_SO_4_. The solvent was removed under reduced pressure. Purification of the crude material was done by column chromatography (CH_2_Cl_2_/methanol = 10:1) (Yield: 0.750 g, 32%). ^1^H NMR (500 MHz, CDCl_3_): δ_H_ (ppm) 2.46 (3H, t, ^4^*J*_HH_ = 2.4 Hz, C≡CH), 2.67 (4H, m, O=C-CH_2_-CH_2_-C=O), 3.40 (3H, s, OCH_3_), 3.66 (180H PEG and 8H C-CH_2_-O, m), 4.13 (6H, d, ^4^*J*_HH_ = 2.4 Hz, O-CH_2_-C≡C). ^13^C{^1^H} NMR (75 MHz, CDCl_3_): δ_C_ (ppm) 29.0, 29.1, 44.0, 63.6, 63.8, 68.6, 68.9, 69.0, 70.5, 71.9, 74.4, 79.7, 171.7, 172.2.

mPEG_2000_-isoxazole

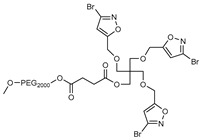



A mixture of mPEG-tripropargylpentaerythritol (0.5 g, 0.00021 mol), ZnCl_2_ (30 mol%) and NaHCO_3_ (0.1 g, 0.00126 mol) in THF (5 mL) was stirred at 45 °C under nitrogen overnight. After completion of the reaction, the solvent was removed under reduced pressure. The crude was extracted three times with CH_2_Cl_2_. After that the organic layer was washed with brine and then dried over anhydrous Na_2_SO_4_. The solid residue after removing solvent in vacuo was purified using a mixture of CHCl_3_/Ether (1:5) to afford the desired pure product with a yield of 0.3 g, 52%. ^1^H NMR (500 MHz, CDCl_3_): δ_H_ (ppm)2.60 (2H, t, ^3^*J*_HH_ = 5 Hz, O=C-CH_2_-CH_2_-C=O), 2.67 (2H, t, ^3^*J*_HH_ = 5 Hz, O=C-CH_2_-CH_2_-C=O), 3.40 (3H, s, OCH_3_), 3.48-4.25 (PEG and 8H C-CH_2_-O, m), 4.57 (6H, s, O-CH_2_-isoxazole ring), 6.36 (3H, s, HC=C). ^13^C{^1^H} NMR (100 MHz, CDCl_3_): δ_C_ (ppm) 28.9, 44.6, 59.0, 62.5, 63.91, 63.94, 69.0, 70.1, 70.3, 70.4, 71.8, 77.2, 106.6, 140.4, 170.8, 171.9, 172.3. ^1^H NMR (500 MHz, D_2_O): δ_H_ (ppm) 2.56 (4H, broad, O=C-CH_2_-CH_2_-C=O), 3.30 (3H, s, OCH_3_), 3.20-4.14 (PEG and 8H C-CH_2_-O, m), 4.50 (6H, broad, O-CH_2_-isoxazole ring), 6.43 (3H, broad, HC=C). ESI-HRMS: m/z = 2710 [M + Na^+^].

### 3.3. Methods

Telodendrimer stock solutions were prepared in MilliQ water (500 µM) and polymer itself was dissolved in DMSO. Different concentrations (1 nM, 1 µM, 10 µM, 15 µM, 25 µM, 50 µM and 100 µM) were prepared by serial dilutions and used to treat cells as described below.

### 3.4. Cell Culture

Human U251N GBM cells were originally obtained from the American Type Culture Collection (ATCC; Rockville, MD, USA). They were cultured in Dulbecco’s Modified Eagle Medium (DMEM; Gibco, Life Technologies Inc. Burlington, ON, Canada) with 5% (*v*/*v*) fetal bovine serum (FBS; Gibco) and 1% penicillin-streptomycin (P/S; Gibco). Cells were incubated at 37 °C with 5% CO_2_ and 95% relative humidity.

### 3.5. Cell Viability Assay

U251N GBM cells were seeded in 96 well plates (5000 cells/well) for 24 h. They were then exposed to different concentrations of the telodendrimer (1 nM, 1 µM, 10 µM, 15 µM, 25 µM, 50 µM and 100 µM) for 24 h or 72 h before being fixed with 4% paraformaldehyde (PFA). The fixed cells were then washed with phosphate buffered saline (PBS) and incubated with 10 µM Hoechst 33342 (Invitrogen, Eugene, OR, USA) for 10 min. This solution was aspirated and replaced with PBS. The cells were then imaged using Leica Fluorescence Microscope (DMI4000B). Images for each condition were analyzed using ImageJ. Cell viability was measured by cell counting. At least one hundred cells were analyzed in 9 random fields per cover slip per condition. Three independent experiments were performed. The cell loss was expressed as % relative to untreated or vehicle (DMSO) treated controls.

### 3.6. Statistics

All data are expressed as mean ± SEM (Standard Error of Mean). Student’s t-tests were used for statistical analyses. P-values less than 0.05 were considered significant. Microsoft Excel’s inbuilt statistical functions were used for all calculations.

## 4. Conclusions

A simple synthetic methodology based on the 1,3-dipolar cycloaddition reaction between an alkyne based core and a bromonitrile oxide, followed by esterification for a stitching hydrophilic polyethylene glycol tail to the core, provides a novel route to architectural polymers containing an isoxazole-based dendron. The addition of a polyethylene glycol tail to the telodendrimer confers solubility in a variety of solvents including aqueous medium, as well as steric stability to the telodendrimer structure [51,52]. These architectural polymers are candidate macromolecular agents exerting marked loss of notoriously resistant glioblastoma cells to therapeutic interventions and our results show that U251N glioblastoma cells are almost completely eliminated when exposed to the telodendrimer. Considering that combination therapy has been proven to be superior to monotherapy for cancer treatment [53], different anti-cancer drugs together with this telodendrimer [25,28] merit further investigations. Certain nanotherapeutics such as liposomal doxorubicin [54] and PTX-loaded human serum albumin nanoaggregates [55] are Food and Drug Administration (FDA)-approved cancer nanotherapeutics. However, their relatively large size (130 nm diameter) restricts tissue penetration and retention. In contrast, telodendrimer based nanoformulations have a smaller size and could potentially be alternative combination anticancer nanotherapeutics. The new methodology presented here provides a versatile platform to expand the scope of isoxazole based systems in nanomedicine. The dendron-linear polymer space in these architectural polymers could be tuned for desired core-corona type assemblies with low critical micelle concentrations. Their potential for combination therapies needs to be further explored by covalent and non-covalent linking of anti-tumor agents, as well as the incorporation of cell surface binding molecular probes for an improved cell-type specificity.

## References

[1] Jensen M.R., Schoepfer J., Radimerski T., Massey A., Guy C.T., Brueggen J., Quadt C., Buckler A., Cozens R., Drysdale M.J. (2008). NVP-AUY922: A small molecule HSP90 inhibitor with potent antitumor activity in preclinical breast cancer models. Breast Cancer Res..

[2] Jancel T., Dudas V. (2002). Management of uncomplicated urinary tract infections. West. J. Med..

[3] Rakesh K.S., Jagadish S., Balaji K.S., Zameer F., Swaroop T.R., Mohan C.D., Jayarama S., Rangappa K.S. (2016). 3, 5-disubstituted isoxazole derivatives: Potential inhibitors of inflammation and Cancer. Inflammation.

[4] Kuz’min V.E., Artemenko A.G., Muratov E.N., Volineckaya I.L., Makarov V.A., Riabova O.B., Wutzler P., Schmidtke M. (2007). Quantitative Structure−Activity Relationship Studies of [(Biphenyloxy) propyl] isoxazole Derivatives. Inhibitors of Human Rhinovirus 2 Replication. J. Med. Chem..

[5] Selvam C., Jachak S.M., Thilagavathi R., Chakraborti A.K. (2005). Design, synthesis, biological evaluation and molecular docking of curcumin analogues as antioxidant, cyclooxygenase inhibitory and anti-inflammatory agents. Bioorg. Med. Chem. Lett..

[6] Chakraborti S., Dhar G., Dwivedi V., Das A., Poddar A., Chakraborti G., Basu G., Chakrabarti P., Surolia A., Bhattacharyya B. (2013). Stable and potent analogues derived from the modification of the dicarbonyl moiety of curcumin. Biochemistry.

[7] Almahariq M., Tsalkova T., Mei F.C., Chen H., Zhou J., Sastry S.K., Schwede F., Cheng X. (2013). A novel EPAC-specific inhibitor suppresses pancreatic cancer cell migration and invasion. Mol. Pharmacol..

[8] Zhu J., Mo J., Lin H.Z., Chen Y., Sun H.-P. (2018). The recent progress of isoxazole in medicinal chemistry. Bioorg. Med. Chem..

[9] Drysdale M.J., Dymock B.W., Finch H., Webb P., McDonald E., James K.E., Cheung K.M., Matthews T.P. (2017). Isoxazole Compounds as Inhibitors of Heat Shock Proteins. U.S. Patent.

[10] FU J., Jin X., Karur S., Lapointe G., Madera A.M., Sweeney Z.K. (2018). Isoxazole Hydroxamic Acid Compounds as LpxC Inhibitors. U.S. Patent.

[11] Fu J.P., Jiang S.Y., Kordikowski A., Sweeney Z.K. (2017). Crystalline Isoxazole Hydroxamic Acid Compounds. U.S. Patent.

[12] Foley Megan A.C., Kuntz K.W., Mills James E.J., Mitchell L.H., Munchhof M.J., Harvey D.M. (2017). Isoxazole Carboxamide Compounds. U.S. Patent.

[13] Duggan M.E. (2017). Deuterated Isoxazole Derivatives and Their Use as Metabotropic Glutamate Receptor Potentiators.

[14] Sun O.Y., Ma J., Wang B., He Y., Xing X., Shen R., Granger B., He J., Long J., Wang G. (2017). Isoxazole Analogs As FXR Agonists And Methods Of Use Thereof. U.S. Patent.

[15] Sun O.Y., He Y., Shen R.C., Xing X., Granger B., Wang B., Ma J., He J., Long J., Wang G. (2017). Isoxazole Derivatives As FXR Agonists And Methods Of Use Thereof. U.S. Patent.

[16] Das A.M., Hazarika M.P., Deka B.P. (2016). Diosgenin Acetate-Isoxazole Derivatives, Process for Preparation Thereof and Their Antifungal Activity.

[17] Kotoku M., Maeba T., Seki N., Hirashima S., Fujioka S., Obika S., Yamanaka H., Yokota M., Sakai T., Hirata K. (2016). Triazole-Isoxazole Compound And Medical USE Thereof. U.S. Patent.

[18] Cole B., Kolodziej A. (2016). Isoxazole Compounds and Methods for the Treatment of Cystic Fibrosis. U.S. Patent.

[19] Yang J., Kopecek J. (2014). Macromolecular therapeutics. J. Control. Rel..

[20] Fenton O.S., Olafson K.N., Pillai P.S., Mitchell M.J., Langer R. (2018). Advances in biomaterials for drug delivery. Adv. Mater..

[21] Sabourian P., Tavakolian M., Yazdani H., Frounchi M., van de Ven T.G.M., Maysinger D., Kakkar A. (2020). Stimuli-responsive chitosan as an advantageous platform for efficient delivery of bioactive agents. J. Control. Rel..

[22] Bodratti A.M., Alexandridis P. (2018). Amphiphilic block copolymers in drug delivery: Advances in formulation structure and performance. Expert Opin. Drug Deliv..

[23] Agrahari V., Agrahari V., Mitra A.K. (2016). Nanocarrier fabrication and macromolecule drug delivery: Challenges and opportunities. Ther. Deliv..

[24] Kakkar A., Traverso G., Farokhzad O.C., Weissleder R., Langer R. (2017). Evolution of macromolecular complexity in drug delivery systems. Nat. Rev. Chem.

[25] Moquin A., Sturn J., Zhang I., Ji J., von Celsing R., Vali H., Maysinger D., Kakkar A. (2019). Unraveling aqueous self-assembly of telodendrimers to shed light on their efficacy in drug encapsulation. ACS Appl. Bio Mater..

[26] Jiang W., Wang X., Guo D., Luo J., Nangia S. (2016). Drug-specific design of telodendrimer architecture for effective doxorubicin encapsulation. J. Phys. Chem. B.

[27] Xiao K., Suby N., Li Y., Lam K.S. (2013). Telodendrimer-based nanocarriers for the treatment of ovarian cancer. Ther. Deliv..

[28] Choi J., Moquin A., Bomal E., Na L., Maysinger D., Kakkar A. (2017). Telodendrimers for physical encapsulation and covalent linking of individual or combined therapeutics. Mol. Pharm..

[29] Zhang X., Huang Y., Zhao W., Chen Y., Zhang P., Li J., Venkataramanan R., Li S. (2014). PEG-farnesyl thiosalicylic acid telodendrimer micelles as an improved formulation for targeted delivery of paclitaxel. Mol. Pharm..

[30] Padwa A., Pearson W.H. (2003). Synthetic Applications of 1,3-Dipolar Cycloaddition Chemistry Toward Heterocycles and Natural Products.

[31] Balalaie S., Shamakli M., Nikbakht A., Alavijeh N.S., Rominger F., Rostamizadeh S., Bijanzadeh H.R. (2017). Direct access to isoxazolino and isoxazolo benzazepines from 2-((hydroxyimino) methyl) benzoic acid via a post-Ugi heteroannulation. Org. Biomol. Chem..

[32] Ladd E., Sheikhi A., Li N., van de Ven T., Kakkar A. (2017). Design and synthesis of dendrimers with facile surface group functionalization, and an evaluation of their bactericidal efficacy. Molecules.

[33] Yazdani H., Bazgir A. (2019). Lewis Acid Catalyzed Regio-and Diastereoselective Synthesis of Spiroisoxazolines via One-Pot Sequential Knoevenagel Condensation/1,3-Dipolar Cycloaddition Reaction. Synthesis.

[34] Sharma A., Mejía D., Regnaud A.l., Uhlig N., Li C.-J., Maysinger D., Kakkar A. (2014). Combined A^3^ coupling and click chemistry approach for the synthesis of dendrimer-based biological tools. ACS Macro Lett..

[35] Sharma A., Kakkar A. (2015). Designing dendrimer and miktoarm polymer based multi-tasking nanocarriers for efficient medical therapy. Molecules.

[36] Moquin A., Sharma A., Cui Y., Lau A., Maysinger D., Kakkar A. (2015). Asymmetric AB3 Miktoarm Star Polymers: Synthesis, Self-Assembly, and Study of Micelle Stability Using AF4 for Efficient Drug Delivery. Macromol. Biosci..

[37] Lam T., Avti P.K., Pouliot P., Tardif J.-C., Rhéaume É., Lesage F., Kakkar A. (2016). Surface engineering of SPIONs: Role of phosphonate ligand multivalency in tailoring their efficacy. Nanotechnology.

[38] Nakahara Y., Kida T., Nakatsuji Y., Akashi M. (2005). New fluorescence method for the determination of the critical micelle concentration by photosensitive monoazacryptand derivative. Langmuir.

[39] Eccles S.A., Massey A., Raynaud F.I., Sharp S.Y., Box G., Valenti M., Patterson L., de Haven Brandon A., Gowan S., Boxall F. (2008). NVP-AUY922: A novel heat shock protein 90 inhibitor active against xenograft tumor growth, angiogenesis, and metastasis. Cancer Res..

[40] Felip E., Barlesi F., Besse B., Chu Q., Gandhi L., Kim S.-W., Carcereny E., Sequist L.V., Brunsvig P., Chouaid C. (2018). Phase 2 Study of the HSP-90 Inhibitor AUY922 in Previously Treated and Molecularly Defined Patients with Advanced Non–Small Cell Lung Cancer. J. Thorac. Oncol..

[41] Kamal A., Bharathi E.V., Reddy J.S., Ramaiah M.J., Dastagiri D., Reddy M.K., Viswanath A., Reddy T.L., Shaik T.B., Pushpavalli S. (2011). Synthesis and biological evaluation of 3,5-diaryl isoxazoline/isoxazole linked 2, 3-dihydroquinazolinone hybrids as anticancer agents. Eur. J. Med. Chem..

[42] Kepp O., Galluzzi L., Zitvogel L., Kroemer G. (2010). Pyroptosis–A cell death modality of its kind?. Eur. J. Immunol..

[43] Aglietti R.A., Dueber E.C. (2017). Recent Insights into the Molecular Mechanisms Underlying Pyroptosis and Gasdermin Family Functions. Trends Immunol..

[44] Kovacs S.B., Miao E.A. (2017). Gasdermins: Effectors of Pyroptosis. Trends Cell Biol..

[45] Dixon S.J., Stockwell B.R. (2019). The hallmarks of ferroptosis. Ann. Rev. Can. Biol..

[46] Dixon S.J. (2017). Ferroptosis: Bug or feature?. Immunol. Rev..

[47] Yang W.S., Stockwell B.R. (2016). Ferroptosis: Death by Lipid Peroxidation. Trends Cell Biol..

[48] Angeli J.P.F., Shah R., Pratt D.A., Conrad M. (2017). Ferroptosis Inhibition: Mechanisms and Opportunities. Trends Pharmacol. Sci..

[49] Stockwell B.R., Friedmann Angeli J.P., Bayir H., Bush A.I., Conrad M., Dixon S.J., Fulda S., Gascón S., Hatzios S.K., Kagan V.E. (2017). Ferroptosis: A Regulated Cell Death Nexus Linking Metabolism, Redox Biology, and Disease. Cell.

[50] Shen Z., Song J., Yung B.C., Zhou Z., Wu A., Chen X. (2018). Emerging Strategies of Cancer Therapy Based on Ferroptosis. Adv. Mater..

[51] Wolfrum C., Shi S., Jayaprakash K.N., Jayaraman M., Wang G., Pandey R.K., Rajeev K.G., Nakayama T., Charrise K., Ndungo E.M. (2007). Mechanisms and optimization of in vivo delivery of lipophilic siRNAs. Nat. Biotechnol..

[52] Li Z., Tan S., Li S., Shen Q., Wang K. (2017). Cancer drug delivery in the nano era: An overview and perspectives. Oncol. Rep..

[53] Mokhtari R.B., Homayouni T.S., Baluch N., Morgatskaya E., Kumar S., Das B., Yeger H. (2017). Combination therapy in combating cancer. Oncotarget.

[54] Franco Y.L., Vaidya T.R., Ait-Oudhia S. (2018). Anticancer and cardio-protective effects of liposomal doxorubicin in the treatment of breast cancer. Breast Cancer.

[55] Ge L., You X., Huang J., Chen Y., Chen L., Zhu Y., Zhang Y., Liu X., Wu J., Hai Q. (2018). Human albumin fragments nanoparticles as PTX carrier for improved anti-cancer efficacy. Front. Pharmacol..

